# Hormonal contraceptive use and risk of pancreatic cancer—A cohort study among premenopausal women

**DOI:** 10.1371/journal.pone.0206358

**Published:** 2018-10-30

**Authors:** Sedrah Arif Butt, Øjvind Lidegaardi, Charlotte Skovlund, Philip C. Hannaford, Lisa Iversen, Shona Fielding, Lina Steinrud Mørch

**Affiliations:** 1 Copenhagen University Hospital, The Juliane Marie Centre, Department of Gynecology, Copenhagen, Denmark; 2 Academic Primary Care, Institute of Applied Health Sciences, University of Aberdeen, Aberdeen, United Kingdom; 3 Medical Statistics Team, Institute of Applied Health Sciences, University of Aberdeen, Aberdeen, United Kingdom; Seoul National University, REPUBLIC OF KOREA

## Abstract

**Importance:**

The association between the use of hormonal contraceptive and pancreatic cancer among premenopausal women has until now been unclear. This is the first study to investigate the risk of pancreatic cancer in pre-menopausal women.

**Objective:**

To determine whether hormonal contraception increases the risk of developing pancreatic cancer in pre-menopausal women.

**Design:**

A nationwide prospective cohort study followed all women in Denmark in the age range of 15–49 years without previous cancer or venous thrombosis from 1995 to 2014. The Danish National Prescription Registry provided individually updated exposure information on use of hormonal contraception. The Danish Cancer Registry provided cancer diagnoses, and the Danish National Patient Register containing clinical diagnoses and surgical codes at discharge from public and private hospitals.

**Setting:**

Population-based cohort study.

**Participants:**

All women living in Denmark aged 15–49 years at January 1st, 1995, and those subsequently reaching age 15 years up to December 31st, 2014 were eligible for the study.

**Results:**

Among 1.9 million women who were followed on average for 11.4 years, 235 pancreatic cancers occurred. Compared to never users, ever users of any type of hormonal contraception had a relative risk (RR) of pancreatic cancer of 0.90 (95% confidence interval (CI) 0.68–1.19). No overall association between duration of hormonal contraceptive use and pancreatic cancer risk was found. Neither was long-term use of hormonal contraception associated with pancreas cancer, RR 0.83 (95% CI 0.47–1.50). The risk did not vary between users of combined and progestogen-only products. All models were adjusted for age, completed or ongoing education, polycystic ovary syndrome, endometriosis and among parous women; parity, age at first birth, smoking and body mass index.

**Conclusions and relevance:**

Compared to never users the risk of pancreatic cancer is not significantly higher among current and recent users of contemporary hormonal contraception and does not vary between users of combined and progestogen-only products. In conclusion, our study suggests no risk of pancreatic cancer with use of any type of hormonal contraception.

## Introduction

The worldwide incidence rate of pancreatic cancer is 10.9 per 100,000 women versus 13.9 per 100,000 men; and pancreatic cancer is relatively rare in young women [1]. However, the mortality rate of pancreatic cancer is almost identical with its incidence rate [2], reflecting its high case-fatality rate.

Sex-steroid hormone receptors have been detected in both normal and neoplastic human pancreatic tissue [3]. Estrogen has been found to inhibit the growth of pre-neoplastic pancreatic lesions or transplanted pancreatic carcinoma in rats and progesterone may possess anti-neoplastic properties [3]. The pancreas is not necessarily considered a target organ of sex steroids, but various clinical and experimental findings demonstrate effects of progesterone on pancreatic endocrine function due to progesterone receptors expressed in islet cells; and reproductive factors possibly play a role in the etiology of pancreatic cancer. [4][5]. Today, approximately 140 million women worldwide or 13% of women aged 15–49 years use hormonal contraception.[6] Thus, determining the relationship between use of hormonal contraception and pancreatic cancer is important given the high prevalence of hormonal contraceptive use and the high case-fatality rate of pancreatic cancer. A majority of previous studies on hormonal contraception and pancreatic cancer have been case-control and cohort studies, and report no association between use of oral contraception and risk of pancreatic cancer. [7–10][11–13][14–16][17]. However, one prospective cohort study of nearly 120,000 women followed over 14 years found that long-term users of hormonal contraception had a, statistically significant, 72% increased risk of pancreatic cancer compared to never use [3]. In this study, oral contraception use was self-reported via questionnaires. Moreover, the inclusion of postmenopausal women in the study population could cause bias with regards to the risk of pancreas cancer due to the use of postmenopausal hormone therapy (HT). Some studies suggest that use of HT decreases the risk or has no association to the risk of developing of panceatic cancer.[3,12][17] Other studies suggest that postmenopausal women alone are at increased risk of pancreatic cancer. [11] To ensure no bias caused by factors related to the postmenopausal age, we investigated the use of different types of contemporary hormonal contraception and the risk of pancreatic cancer in a large cohort of pre-menopausal women.

## Materials and methods

### Data sources

Since 1960 all citizens in Denmark have had a personal identification number recorded in the Civil Registry that holds records of age, death, and emigration. Data from The Danish Cancer Register (containing histologically verified cancers since 1943) provided information on any malignant disease; the Danish National Patient Register contains clinical diagnoses and surgical codes at discharge from public and private hospitals since 1976, and the National Birth Register information about births since 1973. The National Register of Medicinal Product Statistics contains details of redeemed prescriptions for hormonal contraception and was linked using the personal identification number as the key identifier. Data from the National Register of Medicinal Product Statistics is considered complete from January 1, 1995 and this was consequently the start date of this study. Data was stored and analyzed on servers at Statistics Denmark. The unique personal identification numbers were encrypted ensuring anonymity during the data analyses.

### Study population

All women living in Denmark aged 15–49 years at January 1st, 1995, and those subsequently reaching age 15 years up to December 31st, 2014 were eligible for the study.

### Hormonal contraception

From the National Register of Medicinal Product Statistics, we obtained information on the date of redeemed prescriptions, the specific Anatomical Therapeutic Chemical code, dose, number of packages, defined daily doses, and route of administration (tablet, patch, IUD, etc.). The use of hormonal contraception was updated daily, with women changing status at time of cessation or switch in type of hormonal contraception used. Gaps between prescriptions of less than 28 days were filled out prospectively.[18]

Hormonal contraceptive use was categorized as current or recent use (cessation within last six months), previous use (cessation more than six months ago) or never use. The levonorgestrel-intrauterine system (IUD) was used for four years, unless pregnancy or another hormonal product was prescribed before the IUD expired.

### Confounding factors

The National Birth Register and Statistics Denmark provided information on potential confounders; parity (0, 1, 2, 3, 4 or >4), age at first birth among parous women (<25, 25–30, >30 years), smoking status among parous women (yes, no, unknown; available since 1991), body mass index among parous women (<18.5, 18.5–25, 25.1–30, >30, unknown; available since 2004) and completed or ongoing education (elementary school only, high school only, further education but not college/university, college/university education, university education with research qualifications, unknown). The Danish National Patient Register provided information on diagnosis of polycystic ovary syndrome (yes/no) or endometriosis (yes/no). All models were adjusted for these conditions since women with these diseases more often use hormonal contraception.

### Analysis

Data was analyzed by Poisson regression using SAS statistical software, version 9.1 (SAS Institute, Inc., Cary, North Carolina) to calculate incidence rate ratios (referred to as relative risk) and 95% confidence intervals. Age was used as a timescale in the Poison regression analyses with 5-year age bands. The study population was followed until a first diagnosis of pancreatic cancer, death, emigration, 50 years of age, or end of follow-up on December 31, 2014, whichever came first. Women were censored permanently at time of cancer diagnosis, infertility treatment, or venous thrombosis. Women were censored temporarily during pregnancy and for six months after delivery.

The following time-dependent variables were updated daily: hormonal contraception variables, age, calendar year, parity, age at first birth, and education. The simple adjusted models included hormonal contraception, age, and calendar year. Calendar year was included to account for possible changes in the use of specific types of contraception and in pancreatic cancer incidence during the study period. The fully adjusted models included all the time-dependent and the time-constant variables; polycystic ovary syndrome (ever), and endometriosis (ever). Additional adjustment for body mass index and smoking status and the time of first birth was conducted among parous women with this information. The reference group in each analysis was women who had never used hormonal contraception at a given time during follow-up. Calculated risk ratios will be referred to as relative risks throughout the manuscript, this includes all tables.

A search of the PubMed database was performed where relevant articles were identified using the following search terms: “pancreatic cancer”, “pancreatic adenocarcinoma”, “pancreatic neoplasm” combined with “reproductive factors”, “oral contraception”, “exogenous hormone use”. Date of last search was on the 1^st^ of February 2018. A flow chart further describing the inclusion of studies can be found in the supporting information. (S1 Fig).

### Approval

Data was made available through Statistics Denmark and approval obtained from the Danish Data Protection Agency and Health Data Board (previous National Board of Health). According to the Danish Research Ethics Committee Law (§ 8, section 3), ethical approval is not required for register-based studies. Data must be requested from the Danish National eHealth Authority due to legal restrictions on sharing data publicly. Data requests are sent through the application form at: https://sundhedsdatastyrelsen.dk/da/forskerservice/ansog-om-data (the website is in Danish).

## Results

From 1995 to 2014 women aged 15–49 years old accumulated 12.9 million person-years of hormonal contraceptive use and 235 pancreatic cancers incidents were recorded.

Compared to never users, ever users of any type of hormonal contraception had a relative risk of pancreatic cancer of 0.90 (95% confidence interval (CI) 0.68–1.19) (Table 1).

**Table 1:** Relative risk of pancreatic cancer in premenopausal women according to length of hormonal contraceptive use.

Hormonal Contraception	Person years	Cancer events	Relative risk*	95% Confidence Interval	
Never use	8,109,998	139	1	Reference	
Ever use	12,905,304	115	0.90	0.68	1.19
Previous use (>6 months ago)	4,873,757	58	0.80	0.57	1.13
Current or recent use	8,031,547	57	1.01	0.72	1.42
**Duration of current use**					
< 5 years	4,391,494	26	1.21	0.78	1.88
5–10 years	2,434,211	16	0.91	0.53	1.57
>10 years	1,205,842	15	0.83	0.47	1.50
**Time since last use**					
< 5 years	3,296,650	27	0.74	0.48	1.13
5–10 years	1,109,695	20	0.93	0.57	1.53
>10 years	474,230	11	0.78	0.41	1.51

* Adjusted for age, year, education, PCOS, endometriosis, and parity.

Current or recent users of any type of hormonal contraception had a relative risk of 1.01 (95% CI 0.72–1.42). No overall association between duration of hormonal contraception use and pancreatic cancer risk was found (Table 1). Neither was long-term use (more than 10 years) of hormonal contraception associated with cancer of the pancreas: relative risk 0.83 (95% CI 0.47–1.50). Compared to never use, former users had a relative risk of 0.80 (95% CI 0.57–1.13), not significantly changing with time since latest use (Table 1).

Compared to never users, current or recent use of progestin only contraception showed a relative risk of 1.16 (95% CI 0.79–1.89) and combined oral contraception a relative risk of 0.92 (95% CI 0.62–1.36) (Table 2). The simple and fully adjusted results were almost identical. Additional adjustment for smoking status and body mass index in parous women did not change the estimates.

**Table 2:** Relative risk of pancreatic cancer in users of different types of hormonal contraception.

Hormonal contraception	Person-years	Cancer events	Relative risk*	95% Confidence Interval	
Never use	8,109,998	139	1	Reference	
Oral combined 20–40 ug EE	6,915,192	36	0.92	0.62	1.36
Progestin only	994,686	21	1.16	0.71	1.89

*Adjusted for age, year, education, PCOS, endometriosis, and parity.

## Discussion

In accordance with most of the current evidence we found no association between hormonal contraception use and pancreatic cancer risk [9–13,17,19,20](S1, S2 and S3 Tables). Neither was long-term use associated with risk of pancreas cancer which is in contrast to the suggestion by one previous cohort study [3].

The exposure of both exogenous estrogens and endogenous estrogens (i.e. parity and menopause) and risk of pancreatic cancer have been explored in epidemiological studies and results are not consistent [8][19]. A systematic review of several case-control and cohort studies concludes, that reproductive factors have no association with the development of pancreatic cancer in women [19]. However, anti-estrogens such as tamoxifen have been observed having an inhibitory effect on the early stages of pancreatic carcinogenesis [21]. Some cases of unresectable liver metastasis of pancreatic cancer have shown favorable prognosis after being treated with tamoxifen, an anti-estrogen drug. These were solid pseudopapillary neoplasms a low-grade malignant pancreatic tumor comprising 0.9% to 2.7% of all pancreatic malignancies. Solid pseudopapillary neoplasms occur predominantly in premenopausal women and therefore female sex-hormones have been presumed to be associated with the development and growth of these tumors. However, clinical trials with tamoxifen in pancreas cancer have not shown therapeutic effect [19].

No prior study has assessed the risk of pancreatic cancer with progestin-only products in pre-menopausal women. We found no influence on risk of pancreas cancer with use of these products.

### Limitations

We were not able to adjust for alcohol consumption and dietary factors [19][22]. If women on hormonal contraception have higher alcohol consumption than never-users, we would have overestimated the risk of pancreas cancer associated with current or recent use of hormonal contraception. However, since our risk estimates were not adjusted for these factors, hormonal contraceptive use is unlikely to increase the risk of pancreas cancer; if anything, these factors might have biased a potential decreased risk with hormonal contraception use. We cannot be sure that residual confounding or unknown confounders could influence the results. If our results are biased it would most likely imply that we had failed to notice a protective effect of hormonal contraception which would be contradictory to other studies which have explored the same association.[23][11][10] Finally, redeemed prescriptions of medication are not necessarily taken. Repeat prescriptions account for of most of the exposure history, and thus this potential misclassification of exposure is unlikely to have influenced our results.

### Strengths

Our study adds to the sparse evidence on the influence of newer hormonal contraceptive products on the pancreatic cancer risk in women of reproductive age. Our nationwide study is a large-scale prospective cohort study with 1.9 million Danish women followed for an average of more than ten years. We had complete follow-up until diagnosis of cancer, venous thrombosis, infertility treatment, emigration, death or end of study. The validity of pancreatic cancer diagnoses is high because the Danish Cancer Register cross-check their records with histology records to ensure both a high level of completeness and validity of diagnoses. [24,25][26]

The validity of exposure is also likely to be high in our study because recall bias has been eliminated. Information about redeemed hormonal contraception prescriptions is transferred electronically from the pharmacies using bar codes. Thus, we were able to account for switch in use and cessation of use. Our information about both exposure and relevant confounders was updated daily. Information about hormonal contraception and pancreatic cancer was recorded in the relevant national registers without the intention of exploring the association between hormonal contraceptive use and clinical outcomes known, making differential ascertainment of exposure and cancer incidence unlikely.

Our results were adjusted for age, year, educational attainment, number of births, endometriosis, and polycystic ovary syndrome. Additionally, for parous women, smoking and BMI. Adjustment for smoking and BMI did not change our estimates noticeably indicating no major confounding influence of these factors.

The novelty of this study is based upon the quality and quantity of our data utilized for our analysis. This dataset is unprecedented compared to other studies in this area and our focus has solely been on premenopausal women. It is reassuring that we do not find an increased risk of pancreatic cancer in premenopausal women using hormonal contraceptives due to the serious nature of the disease and high mortality rate.

## Conclusion

In conclusion our study suggests no risk of pancreatic cancer with use of any type of hormonal contraception in premenopausal women. Thus, the risk of pancreatic cancer is not a factor to be considered when assessing the risks and benefits of hormonal contraceptive use.

## Supporting information

S1 Fig Flow chart of inclusion of studies. (DOCX)

S1 Table Case-control studies. Studies assessing the risk of pancreatic cancer in users of hormonal contraception stratified according to study design as case-control studies.
(DOCX)

S2 Table Cohort studies. Studies assessing the risk of pancreatic cancer in users of hormonal contraception stratified according to study design as cohort studies.
(DOCX)

S3 Table Meta analyses. Studies assessing the risk of pancreatic cancer in users of hormonal contraception stratified according to meta analyses.
(DOCX)
