# Correction: Hormonal contraceptive use and risk of pancreatic cancer—A cohort study among premenopausal women

**DOI:** 10.1371/journal.pone.0214771

**Published:** 2019-03-28

**Authors:** Sedrah Arif Butt, Øjvind Lidegaard, Charlotte Skovlund, Philip C. Hannaford, Lisa Iversen, Shona Fielding, Lina Steinrud Mørch

The second author’s name is spelled incorrectly. The correct name is: Øjvind Lidegaard. The correct citation is: Butt SA, Lidegaard Ø, Skovlund C, Hannaford PC, Iversen L, Fielding S, et al. (2018) Hormonal contraceptive use and risk of pancreatic cancer—A cohort study among premenopausal women. PLoS ONE 13(10): e0206358. https://doi.org/10.1371/journal.pone.0206358.
