# Supplementary material for: Hormonal contraceptive use and risk of pancreatic cancer—A cohort study among premenopausal women
Source: PLoS One. 2018 Oct 30;13(10):e0206358. doi: 10.1371/journal.pone.0206358 (PMC6207333; doi:10.1371/journal.pone.0206358)
Supplement: S1 Fig — (DOCX) [file pone.0206358.s001.docx]

***Figure 1***

*Flow chart of inclusion of studies*
