# Supplementary material for: Hormonal contraceptive use and risk of pancreatic cancer—A cohort study among premenopausal women
Source: PLoS One. 2018 Oct 30;13(10):e0206358. doi: 10.1371/journal.pone.0206358 (PMC6207333; doi:10.1371/journal.pone.0206358)
Supplement: S1 Table — Studies assessing the risk of pancreatic cancer in users of hormonal contraception stratified according to study design as case-control studies. (DOCX) [file pone.0206358.s002.docx]

**S1 Table**

***Case-control studies***

| **First author,  year,  country** | **Study period** | **Study design** | **Cases/ controls** | **Age group*** | **Adjusted for** | **HC exposure Results Odds ratio** |
| --- | --- | --- | --- | --- | --- | --- |
|  |  |  |  |  |  |  |
| Ji, | 1990- 1993 | Case- | 183/ 1552 | 20 - 55+ | Age, income, education, smoking, age at first birth. | Ever use OR 1.78  (0.91-3.47) |
| 1996 |  | control |  |  |  |  |
| China |  | PB |  |  |  |  |
| Kreiger, | 1995- 1996 | Case- | 52/ 233 | 25 – 74 | Menarche, menopause, HT, OC, age, smoking status, BMI, tofu, and dietary fat. | Use >6 mts OR 0.55  (0.25–1.19) |
| 2001 |  | control |  |  |  |  |
| Canada |  | PB |  |  |  |  |
| Duell, | 1995- 1999 | Case- | 102/ 818 | 21 – 85 | Menarche, menopause, hysterectomy, BO, HT, OC, age, education and smoking. | Ever use OR 0,95  (0.65-1.40) |
| 2005 |  | control |  |  |  |  |
| USA |  | PB |  |  |  |  |
| Duell, | 1983- | Case- | 47/ 821 | All ages | Menarche, menopause, hysterectomy, BO, HT, OC, age, smoking, and schooling. | Ever use OR 0.74  (0.43–1.26) |
| 2009 | 1988 | control |  |  |  |  |
| Multicentre |  | PB |  |  |  |  |
| Zhang, | 1976- | Case- | 17/ 1096 | 23 – 78 | Menarche, menopause, HT, OC, age, race, year, education, BMI, smoking, alcohol, DM, center. | Use >10 yrs OR 2.0 (1.0–4.0) |
| 2010 | 2006 | control |  |  |  |  |
| USA |  | HB |  |  |  |  |
| Lucenteforte, | 1983- | Case- | 20/ 713 | < 80 | Menarche, menopause, BSO, HT, hysterectomy, OC, age, education, residence, year, DM, smoking. | Ever use OR 1.04 (0.55-1.98) |
| 2011 | 2009 | control |  |  |  |  |
| Italy |  | HB |  |  |  |  |

*PB: Population based, HB: Hospital based, BO: bilateral oophorectomy, BMI: Body Mass Index,
DM: Diabetes, OC: oral contraceptive use, HT: Hormone Therapy, IUD: Intrauterine device,
OR: Odds ratio*

****No studies provided estimates for women under 50 years of age.***
