# Supplementary material for: Hormonal contraceptive use and risk of pancreatic cancer—A cohort study among premenopausal women
Source: PLoS One. 2018 Oct 30;13(10):e0206358. doi: 10.1371/journal.pone.0206358 (PMC6207333; doi:10.1371/journal.pone.0206358)
Supplement: S2 Table — Studies assessing the risk of pancreatic cancer in users of hormonal contraception stratified according to study design as cohort studies. (DOCX) [file pone.0206358.s003.docx]

**S2 Table**

***Cohort studies***

| **First author,  year,  country** | **Study** | **Study** | **Events/** | **Age group*** | **Adjusted for** | **HC exposure Results HR, RR or OR** |
| --- | --- | --- | --- | --- | --- | --- |
|  | **period** | **design** | **Obs years** |  |  |  |
| Navarro, | 1998- | Cohort | 102/ 89,835 | 40 – 59 | Menarche, HT, OC, age, smoking, height, study center. | Ever use HR 1.09 (0.81–1.48) |
| 2005 | 2000 |  |  |  |  |  |
| Canada | 16,4 |  |  |  |  |  |
| Skinner, | 1976- | Cohort | 83/ 115,447 | 30 – 55 | Menarche, menopause, HT, OC | Ever use RR 1.21 (0.91–1.61) |
| 2003 | 1998 |  |  |  |  |  |
| USA | 22 |  |  |  |  |  |
| Teras, | 1982- | Cohort | 29/ 287,981 | 51 – 69 | Menarche, age at menopause, hysterectomy, HRT, OC. | Use >11 yrs OR 1.30 (0.90 – 1.90) |
| 2005 | 2000 |  |  |  |  |  |
| USA | 18 |  |  |  |  |  |
| Prizment, | 1986- | Cohort | 228/ 37,459 | 55 – 69 | Age at menarche, age at menopause, hysterectomy, bilateral oophrectomy, HRT, OC. | Ever use HR 0.90 (0.62-1.30) |
| 2007 | 2003 |  |  |  |  |  |
| USA | 18 |  |  |  |  |  |
| Dorjgochoo, | 1997- | Cohort | OC: | 40 – 70 | Menarche, births, menopause, OC, IUD, education, BMI, exercise, smoking, family history of cancer. | Ever use |
| 2009 | 2000 |  | 14/66661 |  |  | OC: 0.8 (0.5-1.6) |
| China | 7,5 |  | IUD: |  |  | IUD: 0.87 |
|  |  |  | 18/66661 |  |  | (0.46-1.63) |
| Duell, | 1992- | Cohort | 304/ 328,610 | 35 – 70 | Menarche, menopause, OC, HT, hysterectomy, BO, age, center, smoking, education, alcohol, meat intake, BMI, DM. | Use >10 yrs HR 1.15 (0.79-1.68) |
| 2013 | 2006 |  |  |  |  |  |
| Europe |  |  |  |  |  |  |
| Lee, | 1995- | Cohort | 47/ 118,164 | 22 – 79 | Mearche, menopause, OC, HT. | Use >10 yrs HR 1.72 (1.19-2.49) |
| 2013 | 2009 |  |  |  |  |  |
| USA | 14 |  |  |  |  |  |
| Kabat, | 1993- | Cohort | 341/ 158,298 | 50 – 79 | Menarche, menopause, OC, HT hysterectomy, BO, age, smoking BMI, education, race, DM | Ever use HR 0.92 (0.80–1.06) |
| 2017 | 1998 |  |  |  |  |  |
| USA |  |  |  |  |  |  |
| Butt, 2018  Denmark | 1995- 2014 | Cohort | 115/ 12,905,304 | 15 – 49 | Age, year, education, PCOS, endometriosis, and parity | Ever use RR 0.90 (0.68 – 1.19) |

*BO: bilateral oophorectomy, BMI: Body Mass Index, DM: Diabetes, OC: oral contraceptive use, HT: Hormone Therapy, IUD: Intrauterine device,
OR: Odds ratio, HR: Hazard ratio, RR: Relative risk,*

****No studies provided estimates for women under 50 years of age.***
