# Supplementary material for: Hormonal contraceptive use and risk of pancreatic cancer—A cohort study among premenopausal women
Source: PLoS One. 2018 Oct 30;13(10):e0206358. doi: 10.1371/journal.pone.0206358 (PMC6207333; doi:10.1371/journal.pone.0206358)
Supplement: S3 Table — Studies assessing the risk of pancreatic cancer in users of hormonal contraception stratified according to meta analyses. (DOCX) [file pone.0206358.s004.docx]

**S3 Table**

***Meta analyses***

| **First author,  year,  country** | **Study** | **Study** | **Events/** | **Age group*** | **Adjusted for** | **HC exposure Results HR, RR or OR** |
| --- | --- | --- | --- | --- | --- | --- |
|  | **period** | **design** | **Obs years** |  |  |  |
| Lujan-Barroso, 2016 | Meta analysis | Meta | 660/ 4748 | All ages | Menarche, menopause, BO, HT hysterectomy, OC, education, BMI, smoking, DM, alcohol, and center. | Ever use OR 0.83 (0.69–1.01) |
|  |  | analysis |  |  |  |  |
| Tang, 2016 | Meta analysis | Meta analysis | 27 studies involving  > 2,300,000 subjects | All ages | Maximum number of confounders were utilized; when unavailable, raw data were used | Ever use 1.09 (95% CI 0.96–1.23) |

*BO: bilateral oophorectomy, BMI: Body Mass Index, DM: Diabetes, OC: oral contraceptive use,
OR: Odds ratio, HR: Hazard ratio, RR: Relative risk,*

****No studies provided estimates for women under 50 years of age.***
